# Determinants of drug expenditure in the Swiss healthcare market in 2006

**DOI:** 10.1186/s12913-022-08212-x

**Published:** 2022-07-07

**Authors:** Yves Eggli, Anne Decollogny, Romain Piaget-Rossel, Patrick Taffé

**Affiliations:** Center for Primary Care and Public Health (Unisanté) – University of Lausanne, Route de la Corniche 10, 1010 Lausanne, Switzerland

**Keywords:** Expenditure, Drug market, Switzerland, Multilevel model, Bayesian analysis, Concurrence

## Abstract

**Background:**

Several measures are in force in Switzerland to control the cost of drugs, but are not effective enough. There are many determinants influencing these expenditures, related to treatments, markets, physicians, patients and regions, but their impact on costs is not clear.

**Methods:**

We applied a Bayesian multilevel model with five levels to adjust for patients, drugs’ market, and physicians ‘characteristics, treatment type, and district (i.e. Swiss canton). We used data of the Swiss drugs’ market in 2006, offering real choices for doctors and patients (multiple products for similar active substances), with a neutral position of pharmacists (no financial incentives).

**Results:**

Variance partitioning of yearly drugs’ cost per insured showed that market level (delivered substance) contributed to 76% of the variance, treatment level (delivered product) to 20%, whereas patients’ and physicians’ levels accounted for only 2% each, without significant differences between Swiss cantons. After adjusting for covariables at each level, the model explained about 51% of the variation at the market and 20% at the treatment levels. We found that older but substitutable drugs, generics, larger size of the market and physician’s specialty were associated with lower expenditure, whereas drugs requiring a physician’s prescription, the number of prescribers per patient, patient’ age, male gender, and comorbidities increased expenditure. Our results show that for a specific medication the yearly cost of recently released drugs was 36 CHF higher than for similar and substitutable drugs introduced 15 years earlier, corresponding to one third of the average annual treatment cost observed in our dataset. Competition did not seem to be effective to reduce expenditure on the drug market.

**Conclusion:**

The main finding of this study is that recentness of drugs was associated with an increase in drug expenditure in 2006, even after adjustment for all non-controllable determinants. Further research is recommended to confirm those results with updated data.

## Background

In 2018, drugs were sold for CHF 9.5 billion (public prices) in Switzerland [1], which represented 11.8% of total health costs (CHF 80.2 billion) and CHF 1,108 per inhabitant. In terms of ex-factory prices, the annual turnover of drugs rose from CHF 3.3 to CHF 5.0 billion between 2006 to 2018 [2], corresponding to an increase in ex-factory cost of drugs per capita of 33.5%, while the inflation was 2.5% during the same period [3] (Table 1). A part of this increase was related to a growth in the number of prescribed packaging per capita (+ 7.4%), but a higher part was due to the ex-factory price increases (+ 24.3%). This price driven contribution to drugs expenditure was also observed in other countries [4].

**Table 1:** Increase of drugs’ costs from 2006 to 2018

Indicators	2018	2006	Difference	Growth
Number of delivered packages (millions)	126.4	103.1	23.3	22.6%
Annual turnover^a^ (Swiss francs, millions)	5 034	3 303	1 731	52.4%
Average cost per package^a^ (Swiss francs)	39.8	32.0	7.8	24.3%
Population (millions)	8.545	7.484	1.061	14.2%
Per capita number of delivered packages	14.79	13.78	1.02	7.4%
Per capita cost of drugs^a^ (Swiss francs)	589.1	441.4	147.8	33.5%

^a^Ex-factory prices

Several measures to curb drug expenditures are in force in Switzerland since 2001. First, patients have to pay the full cost until they reach a certain deductible, set by contract with their health insurer [5]. When they reach their deductible, patients still have to pay a 10% share up to a certain ceiling. Second, pharmacists receive an administrative tax for delivering drugs to reduce the perverse incentive of a remuneration proportional to the net sales [6]. Third, pharmacists are allowed to substitute medical prescriptions for the cheapest products if there are generic drugs [7]. Fourth, the prices of drugs are set administratively by the Confederation (Federal Office of Public Health, FOPH) on the basis of legal criteria (i.e. economics, efficiency, adequacy of the prescriptions according to scientific criteria), after a notice from the Swiss Medicines Commission [8].

The effectiveness of these measures was however criticized [9], and given the strong and continuing rise in the drug expenditure in Switzerland, politicians are willing to understand determinants of this rise in order to design appropriate measures that would limit it.

In 2017, the Swiss Federal Council commissioned a panel of expert to tackle the issue of costs increase in compulsory health insurance [10]. Among the 37 measures proposed in their report, four might help reducing drug expenditure. In August 2019, the Federal Council proposed to the Swiss Parliament to implement a first set of nine measures, including one related to drug pricing. Such a measure is applied in Europe [11], and consists in introducing a reference price for three kinds of drugs: generic drugs (i.e. same active substance as the original drug available when the patent has expired), "me-too" (i.e. different active substances with the same mechanism of action as the original drug), and some patent-protected drugs that are not very innovative. For drugs whose price is higher than the reference price, the delta would be at the expense of the patient. However, the motion currently (2022) discussed by the Swiss Parliament is limited to generics. In order to determine whether imposing a reference price to other substances (i.e. me-too and patent-protected drugs) is necessary, one might be willing to look at the impact of the age of drugs (i.e. the time since they have been introduced on the Swiss reimbursement list) on their prices. Indeed, if products introduced recently, by stimulating competition, reduces drug prices, extending reference prices to other substances (“me-too”) is not necessary. On the opposite, it might be wise to recommend this extension if medicinal products introduced recently tends to increase expenditure.

From a theoretical economic point of view, the first assertion should be observed in a perfect competition market [12]. Nevertheless, there is some evidence that the drug market is imperfect [13]. It is thus important to analyze empirically the determinants of drug retail expenditure, which we did in this study. Our objective was to analyze the possible impact of several variables on different levels: market (number of treated patients, competition for instance), physician (specialist or not), patients (age, gender, deductible, co-morbidities), regions (cantons), and the treatments (generics for instance), with, for every level, a special attention paid to the recentness of drugs (i.e. minus the age of drugs).

A recent literature review has shown a significant effect of the expiry date of patents, the access to the generic market, and it emphasized the importance of tendering [14]. However, this review paper also indicates that scientific knowledge on the determinants of drugs expenses is lacking, largely related to a lack of transparency on this particular market. What is more, in Switzerland it is difficult to obtain representative drug data from insurers because such data could reveal the risk structure of insured and harm their risk financial compensation interests. Our study is based on 2006 data that four major insurance companies accepted to provide, anonymously, thanks to the support of the Federal Office of Public Health [15]. Although these data are arguably old, they provide a valuable and very rare source to understand the functioning of the Swiss drugs’ market, while presenting some important advantages: they are highly representative of the Swiss population and they describe a drug market offering a significant choice among multiple products (18 per specific market in average) at a time when the generics were significantly gaining importance.

## Methods

### Studied population

Our source population came from four sickness funds and consisted in 473,886 insured living in Switzerland in 2006. Those data were already used to study the determinants of new drugs prescription in Switzerland [15], where it was shown that they were remarkably representative of the demographic structure of the population. In 2006, pharmacists already had a more neutral role in the choice of products, because they did no longer receive a percentage of the selling price but an administrative tax. Moreover, this year was a relatively stable year for regulated prices, whereas generics represented a growing share of the drug market (i.e. 47.4% of the potential marketed drugs with no more patent protection at that time, measured in number of outpatients drugs’ packages) [16]. The setting was a random sample of 100,000 insured living in the nine cantons prohibiting doctors’ drugs delivery (Aarau, Basel-Stadt, Fribourg, Geneva, Jura, Neuchâtel, Ticino, Valais and Vaud). We made this choice to ensure the exhaustiveness of drugs’ deliveries, because pharmacists sent systematically drugs’ claims to insurers but not physicians. Bills provided detailed information on active substances (medicinal product) and marketed package (i.e. its brand name, producer, formulation, pharmaceutical form, dosage, and quantity of doses). All data were anonymous, without variables enabling a potential identification of patients or physicians (no birth date or residence data for instance).

### Variables

The dependent variable was the yearly individual (log) treatment cost, i.e. (logarithm of) the total cost of a specific drug given to a patient during the year 2006 (all analyzed drugs belong to the Swiss reimbursement list [17]. As emphasized in the statistical methods section, the dataset exhibited a complex hierarchical multilevel structure with five levels: treatments, markets, physicians, patients, and their cantons. At the lowest level of the hierarchy (i.e. at the level of the observations), lies the treatments prescribed to each individual. Then, the structure is both nested and crossed. Independent variables were measured at each of these levels.

#### Treatment-observation level

A treatment was defined as a given molecule prescribed at least once to a specific patient during the year 2006. If several physicians were involved in the prescription of a given treatment, it gave raise to only one observation. Four variables characterized this level: proportion of generics, proportion of prescription drugs (i.e. drugs requiring a physician prescription, as opposed to over-the-counter drugs), number of physicians seen by the patient who received the treatment, and average age of the drugs prescribed in a given treatment (referred later in the text as “age of drugs”).

When a treatment involved one single prescriber, this yielded a value of 0 or 1 for the variables proportion of generic and proportion of prescription drugs.

#### Market level

The market corresponds to domains in which substitution among substances or products is possible, defined by the fourth level of the Anatomic Therapeutic Chemical (ATC) classification of drugs. Since it corresponds to a specific indication (disease) and a certain mechanism of action, physicians can choose one substance over another within this group. We used six variables to describe markets: size (i.e. number of treated patients on the market), market recentness (i.e. minus mean age of drugs prescribed on the market), the Herfindahl–Hirschman index (HHI) measuring the concentration of suppliers [18], the number of drugs prescribed on the market, the number of active substances (ATC 5^th^ level), and the number of brands.

#### Physician level

Three types of physicians were distinguished: general practitioners (installed as internal medicine or generalists), independent specialists and hospitals specialists (physicians working in a hospital but delivering ambulatory care). Some physicians tend to prescribe more recent drugs than others, regardless of market or patient characteristics. This preference for recent drugs was captured by computing minus the average age of the drugs prescribed by each physician (variable referred to hereafter as “drugs’ recentness preference”).

#### Patient level

We computed following variables to describe patients: five age groups (0–19, 20–39, 40–59, 60–79, 80 +), gender, deductible (lower than 400 Swiss francs or not), and an index of comorbidities (approximated by the number of 3^rd^ level ATC categories).

#### Canton level

Finally, we generated nine dummy variables representing each of the nine cantons.

### Statistical methods

**Fig. 1:**
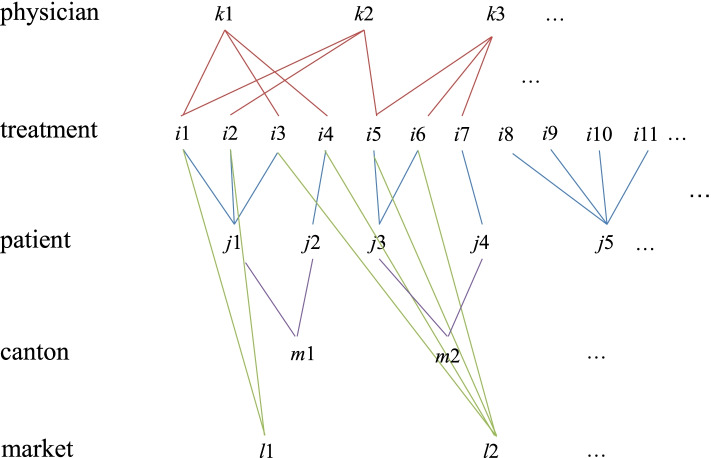
Structure of data

The data have a complex hierarchical multilevel structure exhibiting multiple memberships and cross-classifications [19], which can be represented in Fig. 1:

This figure is self-explanatory and may simply be read as: treatment *i* belonging to drug market *l* and made by physician *k* is addressed to patient *j* in canton *m*.

The dependent variable (i.e. yearly individual treatment cost) was log transformed before analysis as it was extremely skewed. To account for the multiple membership, a counting variable for the number of physicians seen by each patient for each treatment line was generated. The statistical analyses were carried out using the MLwiN package from within Stata [20, 21]. The model was estimated by Bayesian MCMC methods, using a burn-in period of 5,000 iterations followed by a monitoring period of 20,000 iterations. To ensure a decent computing time, our statistical analyses were conducted on a random – hence representative – subsample of 100,000 insured. Independent improper priors were used for the fixed effects and independent hierarchical Normal for the random effects. Diffuse Gamma hyper-priors were specified for precision. The model may be written in hierarchical notations as:

$$y_{ijklm} = \beta_{0} + \beta^{\prime}_{1} x_{ijklm} + u_{j}^{id} + u_{k}^{phys} + u_{l}^{atc5} + u_{m}^{canton} + \varepsilon_{ijklm}$$

$$u_{j}^{id} = \beta_{2}^{id} {\kern 1pt}^{\prime } x_{j}^{id} + \delta_{j}^{id}$$

$$u_{k}^{phys} = \beta_{3}^{phys} {\kern 1pt}^{\prime } x_{k}^{phys} + \delta_{k}^{phys}$$

$$u_{l}^{atc5} = \beta_{4}^{atc5} {\kern 1pt}^{\prime } x_{l}^{atc5} + \delta_{l}^{atc5}$$

$$u_{m}^{canton} = \beta_{5}^{canton} {\kern 1pt}^{\prime } x_{m}^{canton}$$

$$\varepsilon_{ijklm} \sim N(0,\sigma_{obs}^{2} )$$

$$p(\beta_{j} ) \propto 1,\,\,\,j = 0,1,...,5$$

$$p(\delta_{j}^{id} |\sigma_{id}^{2} )\sim N(0,\sigma_{id}^{2} )$$

$$p(\delta_{k}^{phys} |\sigma_{phys}^{2} )\sim N(0,\sigma_{phys}^{2} )$$

$$p(\delta_{l}^{atc5} |\sigma_{atc5}^{2} )\sim N(0,\sigma_{atc5}^{2} )$$

$$p(1/\sigma_{id}^{2} )\sim Gamma(0.001;0.001)$$

$$p(1/\sigma_{phys}^{2} )\sim Gamma(0.001;0.001)$$

$$p(1/\sigma_{atc5}^{2} )\sim Gamma(0.001;0.001)$$

$$p(1/\sigma_{obs}^{2} )\sim Gamma(0.001;0.001)$$

where:

- $y_{ijklm}$ is the logarithm of the yearly cost of a specific treatment given to an individual
- $x_{ijklm}$ is a vector of variables measured at the observation (i.e. treatment) level (available variables: proportion of generics in each specific treatment line, proportion of drugs under prescription in each specific treatment line, number of physicians seen by each patient for each treatment line, average age of the molecules in each specific treatment line)
- $x_{j}^{id}$ is a vector of variables measured at the individual level (available variables: age, gender, deductible ≤ 400 CHF (yes/no), index of comorbidities)
- $x_{k}^{phys}$ is a vector of variables measured at the physician level (available variables: GP/hospital specialist /non-hospital specialist, mean recentness (i.e. minus average age of the drugs prescribed by each physician)
- $x_{l}^{atc5}$ is a vector of variables measured at the ATC (5 characters) market level (available baseline variables: number of insured, market recentness (minus average age of the drugs prescribed); available additional variables: Herfindhal index, number of mee-too, number of drugs available, number of brands available; and cluster means to adjust for confounding by cluster)
- $x_{m}^{canton}$ is a vector of variables measured at the level of the canton (only dummy variables representing each canton were used)

This model can be conveniently rewritten:

$$y_{ijklm} = \mu + \delta_{j}^{id} + \delta_{k}^{phys} + \delta_{l}^{atc5} + \varepsilon_{ijklm}$$

with $$\mu_{x} = \beta_{0} + \beta^{\prime}_{1} x_{ijklm} + \beta_{2}^{id} {\kern 1pt}^{\prime } x_{j}^{id} + \beta_{3}^{phys} {\kern 1pt}^{\prime } x_{k}^{phys} + \beta_{4}^{atc5} {\kern 1pt}^{\prime } x_{l}^{atc5} + \beta_{5}^{canton} {\kern 1pt}^{\prime } x_{m}^{canton}$$.

To compute the mean cost and various contrasts, the following back-transformation will be useful [22]:

$$E({\text{cost}}{\kern 1pt} {|}{\kern 1pt} X{ = }{\kern 1pt} x) = e^{{\mu_{x} + \sigma^{2} /2}}$$ with $$\sigma^{2} = \sigma_{obs}^{2} + \sigma_{id}^{2} + \sigma_{phys}^{2} + \sigma_{atc5}^{2}$$

$$contrast(x) = E({\text{cost}}{\kern 1pt} {|}{\kern 1pt} X{ = }{\kern 1pt} x_{1} ) - E({\text{cost}}{\kern 1pt} {|}{\kern 1pt} X{ = }{\kern 1pt} x_{0} )$$

An unconditional model (i.e. without regressors at the various levels) was estimated to partition the overall variance across the five levels, and the Variance Partition Coefficient (VPC) was computed at each level to assess the proportion of the response variance that lies at each specific level of the model hierarchy [23, 24]. To illustrate the significance of this variance partitioning, based on the empirical Bayes estimates of the random effects, we computed at each level of the hierarchy the contrasts between the percentiles P99 and P1, and P75 and P25, in terms of mean treatment-cost difference. For this, we used the back-transformation to compute the predictions on the original scale.

Then, various conditional models were estimated and the proportion of variation (PEV) explained at each level computed to quantify the contribution of patients’, physicians’, market’s, and treatment’s characteristics to the outcome variance [25]. However, we did not compute the individual variable PEVs as, unless the regressors are all orthogonal, the variable-specific PEVs do not add up to the total level PEV [26]. Instead, to assess the importance of each independent variable we computed the contrast (i.e. the predicted difference in mean outcome values) between two different covariate-patterns. To keep interpretations simple, we contrasted covariate-patterns, which differed by only one variable taking a different value in each pattern (e.g. mean treatment-cost difference between patients in the older and younger age classes or the mean treatment-cost difference between a generic and a non-generic, etc.). Note that the regression coefficients are interpreted as semi-elasticities and, therefore, allow us to compute the percentage change in the outcome value for a unit-change in the regressor value. On the other hand, the back-transformation approach makes it possible to calculate the mean difference of the cost of the treatment in absolute value.

The contrasts were computed by considering all the available independent variables at each level, except at the market-level, where in addition to the two baseline variables (i.e. number of patients and market recentness), only one of the four additional variables (i.e. Herfindhal index, number of mee-too, number of drugs available, number of brands available) was included at a time, as these variables were entangled (and including all of them simultaneously in a model would provide meaningless coefficients). Therefore, we estimated four different regression models, in turn, to compute the impact of these four additional variables. Estimated coefficients of the other variables were quite similar in the four models. In the Result Section, we decided to report those from the model with HHI, since this variable was of particular interest to see whether competition plays its role in the drug market.

At the market level, we adjusted for confounding by cluster by including into each regression model the computed cluster means for the following variables: age of patient, gender, deductible, co-morbidity index, generic, and Rx drug [27, 28].

Proper convergence of the MCMC algorithm was assessed by inspecting the trace plots, smoothed histograms of the posterior distributions, and auto-correlation functions of the parameters. The goodness of fit of the model was assessed by inspecting histograms of the random effects and residuals, as well as scatter plots of residuals versus predicted mean cost of the treatments.

All the analyses were carried out using Stata 15.1 (StataCorp LP, 4905 Lakeway Drive, College Station, TX 77,845, USA) and MLwiN 2.36 (Centre for Multilevel Modelling, University of Bristol).

## Results

### Variables summary

Table 2 describes the different variables used in this study, based on a random sample of about 100,000 insured. More precisely, the setting included 490,197 annual treatments from 328 substitutable markets, prescribed to 99,988 insured, by 9,230 physicians, in 9 different cantons. This means that individual had on average about 5 different treatments (substances) per year. This is representative of the 2006 Swiss drug consumption, knowing that we only took into account drugs from the Swiss reimbursement list dispensed by pharmacists for outpatients and that multiple packages of the same substance were counted only once per patient.

**Table 2:** Descriptions of the variables used

Variables	Study^a^	Switzerland
**Treatments (490,197 unique values)**		
Average yearly individual treatment cost	107 (398)	n.a.^b^
Proportion of generics prescribed	18.09%	n.a
Proportion of Rx prescribed (drug requiring a physicians’ prescription)	76.96%	n.a
Average number of physicians seen by the patient for a specific treatment	1.09 (0.32)	
Average age of drugs	14.98 (11.09)	n.a
**Markets (328 unique values)**		
Average number of treated patients on the market	7160 (13,556)	n.a
Average market recentness	-18.94 (10.31)	n.a
Average HHI value	0.56 (0.26)	n.a
Average number of drugs on the market	18.45 (24.19)	n.a
Average number of active substances on the market	2.82 (2.02)	n.a
Average number of brands on the market	5.62 (5.42)	n.a
**Physicians (9,529 unique values)**		
Proportion of general practitioners	34.28%	n.a
Proportion of independent specialists	62.11%	n.a
Proportion of Hospitals	3.61%	n.a
Recent drugs’ preference (minus average years)	-14.2	n.a
**Patients (99,988 unique values)**		
Age = 0–19 (percentage of patients in the age class)	19.5%	21.7%
Age = 20–39	21.6%	27.0%
Age = 40–59	28.8%	35.1%
Age = 60–79	23.5%	11.6%
Age = 80 +	6.6%	4.6%
Percentage of males	43.2%	49.0%
Percentage of deductibles > 400	34.4%	n.a
Average co-morbidity index values (ATC 3^rd^ level)	4.84 (3.83)	n.a
**Regions (9 unique values)**		
Aarau (patients’ share)	8.73%	19.33%
Basel-Stadt (patients’ share)	4.36%	6.30%
Fribourg (patients’ share)	8.48%	8.62%
Geneva (patients’ share)	26.18%	14.62%
Jura (patients’ share)	0.72%	2.35%
Neuchâtel (patients’ share)	7.26%	5.72%
Ticino (patients’ share)	9.99%	10.94%
Vaud (patients’ share)	25.62%	22.21%
Valais (patients’ share)	8.65%	9.90%

^a^Standard deviation in brackets

^b^n.a. = data not available at the time of our study

Women were overrepresented in our sample (around 57%), while the young age categories were under-represented as compared to the Swiss numbers. More than 34% of the individuals represented in our sample chose a deductible higher than 400 CHF and, in average, they consumed drugs from almost 5 different ATC 3^rd^ level codes (proxy for the number of illnesses).

Independent specialists prescribed more than 55% of the delivered drugs, while less than 10% of the prescriptions were dispensed by physicians working in a hospital (drugs prescribed for ambulatory setting after a hospital discharge, for one-day surgery, or for emergency or planed consultations).

The average age of the drugs prescribed was 14.98 years, 18% of them were generics and 77% were Rx drugs (i.e. drugs requiring a physician’s prescription). These drugs belonged to 328 distinct substitutable markets, which contained around 7,000 insured in average. The markets included 18.45 drugs, 2,82 active substance and 5.62 brands in average.

The average HHI index across these markets was 0.56 while the average number of active substances was almost 3.

Finally, half of the studied patients came from two cantons: Geneva and Vaud, whereas there was an under-representation of the cantons of Aargau, Basel-Stadt and Jura.

### Variance partitioning and contrasts

The partition of the overall variance across the five levels, as well as the contrasts between the percentiles P99 and P1, and P75 and P25 of the empirical Bayes estimates of the random effects are presented in Table 3. The variance partition coefficients (VPC) indicates that most of the response variance lied at the market-level and at the treatment-level (VPC of 76.3% and 19.8% respectively). The contrasts display also much higher ranges for these two levels (P99-P1 larger than 350 Swiss francs for both).

**Table 3:** Unconditional total variance partitioning across the five levels of the model

	Treatment	Market	Patient	Physician	Region
Total VPC (in %)	19.82 [17.47; 22.18]	76.32 [75.51; 79.13]	2.14 [1.88; 2.40]	1.69 [1.47; 1.91]	0.02 [0.00; 0.04]
P99-P1	1274.4	6769.7	181.1	193.2	19.0^a^
P75-P25	158.1	174.4	32.8	40.2	-
PEV (in %)	12.4	50.9	24.8	47.1	-

Confidence intervals in squared brackets. *PEV* Proportion of explained variance. ^a^Min-Max contrast

Looking at the proportion of explained variance allows one to measure the percentage of the variability at a given level that was explained by the variables included at this level in our Model (see Table 4). Variables included at the market and physician levels explained approximately 50% of the cost variability measured at these two levels. Only 12.4% of the cost variability present at the treatment level was explained by the variables included in our model. Given the fact that the VPC at the treatment level was almost 20%, this indicates that unmeasured treatment-level variables might have a significant impact on the treatment cost.

**Table 4:** Estimation of the regression coefficients

Variable	Coefficient	Standard deviation	P-value	95% credible interval
**Treatment (observation)-level**				
Constant	4.44	0.18	0.00	4.15; 4.77
Proportion of Rx drugs prescribed	0.40	0.01	0.00	0.39; 0.41
Proportion of generics prescribed	-0.37	0.00	0.00	-0.38; -0.37
Number of physicians seen by the patient for a specific treatment	0.68	0.00	0.00	0.67; 0.68
Age of drugs	-0.02	0.00	0.00	-0.02; -0.02
Square of average age of drugs	0.12^b^	0.01^b^	0.00	0.10; 0.13
**Market-level**				
Number of treated patients on the market	-0.01^b^	0.00^b^	0.00	-0.01; -0.01
Market recentness	0.05	0.00	0.00	0.05; 0.06
Market concurrence^c^				
HHI	0.08	0.20	0.34	-0.26; 0.45
Number or drugs prescribed on the market	-0.28^a^	0.41^a^	0.21	-0.31; 0.26
Number of active substances	0.04	0.04	0.16	-0.07; 0.10
Number of brands	-0.92^a^	1.5^a^	0.27	-4.8; 1.2
Adjustment for confounding by cluster				
Patient age	-0.43^a^	0.47^a^	0.18	-1.29; 0.66
Male (proportion)	1.29	0.28	0.00	0.73; 1.88
Deductible > 400 CHF (proportion)	0.17^a^	0.10^a^	0.05	-0.02; 0.34
Co-morbidity index	0.15	0.03	0.00	0.09; 0.21
Generics (proportion)	0.25	0.18	0.09	-0.08; 0.56
Rx drugs (proportion)	0.68	0.11	0.00	0.45; 0.93
**Physician-level** (ref.: GP)				
Independent Specialist (0/1)	-0.03	0.01	0.00	-0.04; -0.02
Hospital (0/1)	-0.34	0.01	0.00	-0.37; -0.31
Recent drugs’ preference (years)	0.02	0.00	0.00	0.02; 0.02
**Patient-level** (ref.: Age = 0–19)				
Age = 20–39 (0/1)	0.14	0.01	0.00	0.13; 0.15
Age = 40–59 (0/1)	0.27	0.01	0.00	0.26; 0.28
Age = 60–79 (0/1)	0.35	0.01	0.00	0.34; 0.36
Age = 80 + (0/1)	0.39	0.01	0.00	0.38; 0.40
Male (0/1)	0.04	0.00	0.00	0.03; 0.04
Deductible > 400 CHF (0/1)	-0.01	0.00	0.05	-0.01; 0.00
Co-morbidity index	0.01	0.00	0.00	0.01; 0.01
**Region-level** (ref.: Aarau)				
Basel-Stadt	0.03	0.01	0.01	0.01; 0.05
Fribourg	0.03	0.01	0.00	0.01; 0.05
Geneva	-0.01	0.01	0.06	-0.03; 0.00
Jura	0.04	0.02	0.04	-0.01; 0.08
Neuchâtel	0.02	0.01	0.04	-0.00; 0.04
Ticino	0.03	0.01	0.00	0.01; 0.05
Vaud	-0.01	0.01	0.25	-0.02; 0.01
Valais	0.02	0.01	0.02	0.00; 0.04

^a^result multiplied by 100; ^b^result multiplied by 1000; ^c^only one of the four indexes was used at a time in the regression model

Table 4 displays the results obtained from the estimation of the multilevel regression model with the logarithm of drugs’ cost as the dependent variable. Since the outcome was measured on the natural logarithmic scale, the coefficients multiplied by 100 can be interpreted – *ceteris paribus* – as the percentage changes in treatment cost given a one-unit change in the corresponding covariate (for a proportion this means moving from 0 to 100%).

#### Treatment-observation level

Based on this interpretation, one can conclude that the cost of drugs requiring a physician’s prescription (i.e. Rx drugs) was on average 40% higher than that of over-the-counter drugs *ceteris paribus.* Notice also that markets with a higher proportion of Rx drugs (cluster means) were also associated with higher expenditure (68% more from 0 to 100% Rx prescriptions). The effect of prescribing generics was more contrasted. Indeed, whereas the fact that the prescribed drug was a generic lowered cost at the treatment-level by -37%, *ceteris paribus*, markets with a higher proportion of generics had a higher cost (+ 25%) (although non-significant at the 95% credibility level, credibility interval ranges from -0.08 to 0.56). The annual cost of drugs increased by 68% per additional (per patient) prescriber. Finally, regarding the effect of age of drugs on the cost, as a polynomial of degree two was fitted the coefficients do not have a direct useful interpretation and we refer the reader to Table 5 below for the contrast computed by comparing newly released drugs with fifteen-years-old drugs.

**Table 5:** Contrasts between two different values of the covariate

Variable	Nature of the contrast	Contrast (CHF)
**Treatment-level**		
Rx prescription	Yes – No	92.4 [60.8; 124.0]
Generics	Yes – No	-58.6 [-78.6; -38.5]
Number of prescribers	2 – 1	217.3 [142.9; 291.8]
Age of drugs	15 – 0	-36.2 [-48.6; -23.8]
**Market level**		
Number of insured	100,000—10,000	-108.1 [-145.1; -71.1]
Market recentness	15—0	-104.8 [-140.7; -68.9]
**Physician level**		
Physician’s specialty	Hospital specialist – GP	-54.4 [-73.0; -35.8]
**Patient level**		
Age	(85 +)—(0–19)	88.8 [58.4;119.2]
Male	Male—Female	7.1 [4.7;9.6]
Deductible	(> 400 CHF)—(< = 400 CHF)	-0.96 [-1.3; -0.6]
Co-morbidity index	10—1 illnesses	19.0 [12.5;25.5]
**Region level**		
Canton	Jura—Neuchâtel	3.2 [2.1; 4.3]

95% confidence intervals in squared brackets

#### Market level

Turning to the variables measured at the market level, only two variables displayed statistically significant effects at the 95% credibility level: drugs were significantly cheaper in markets containing a larger number of insured and drugs’ recentness increased average prices on the market. Cluster-mean variables were associated with significant positive coefficients for gender (male), co-morbid index, and Rx prescription.

#### Physician level

With respect to GPs, non-hospital- and particularly hospital- specialists tended to prescribe cheaper drugs’ treatment, *ceteris paribus*.

#### Patient level

Drugs prescribed to old patients and/or to patients with high co-morbidity index were more expensive, *ceteris paribus*. Moreover, drugs prescribed to men were 4% more expensive than drugs prescribed to women. As for drugs prescribed to patients with a higher deductible, they tended to be less expensive.

#### Canton level

Finally, the larger discrepancy between cantons was observed between Geneva and Jura. In the latter drugs prescribed are, in average and *ceteris paribus*, 5% more expensive than in the former.

For all variables with a statistically significant coefficient at the 95% credibility level (except for the cluster means, which were mostly added to adjust for confounding by cluster), we illustrated their impact on drug expenditure by computing the contrast between a low-value covariate-pattern and a high-value one (Table 5). Notice that to compute these contrasts, it is necessary, first, to define a reference covariate-pattern, as the back-transformation is non-linear, and contrasts depend on the covariate values. This reference covariate-pattern was built using the mean of each continuous regressor and the category with the most occurrence for categorical regressors. Notice that the contrasts were computed within levels but not across levels to provide interpretable results.

At the treatment level, the largest contrast resulted from the number of prescribers per treatment, + 217 CHF for treatments prescribed by 2 vs 1 prescriber. The prescription of similar but older drugs (drugs’ substitution) allows one to reduce expenditure by -36.2 CHF for drugs of average age 15 years older. The annual cost of Rx drugs was 92.4 CHF higher than that of over-the-counter drugs, while generic drugs were 58.6 CHF cheaper than non-generic ones.

At the market level, drugs prescribed on larger markets were cheaper on average (i.e. -108 CHF for markets with 100,000 insured vs 10,000). The contrast between a market with new drugs vs a market with average drug age 15 was 104.8 CHF, showing that on younger markets drugs are more expensive than older ones (notice that these markets may not be substitutable as they are selling different drugs).

At the physician level, prescriptions from specialists were cheaper than GPs’ (-54 CHF). This result might seem counterintuitive since specialists are expected to prescribe more costly drugs. In fact, they do prescribe more expensive molecules but adjusting for the prescribed molecules as we did in our model (via the market adjustment) turns out to reverse this trend.

At the patient level, drugs prescribed to people with 10 illnesses vs 1 were 19 CHF more expensive, and drugs prescribed to old people (85 years old or more) were 88.8 CHF more expensive than drugs prescribed to children and teenagers.

All remaining variables with a significant coefficient (i.e. male, deductible, and canton) had a low impact on drug expenditure.

## Discussion

In 2016, drug expenditure represented 11.8% of the total health costs in Switzerland. While several measures are being proposed to curb these ever-growing costs, we found it interesting to propose an overview of the likely determinants of drug expenditure and to assess their relative impact. We started by partitioning the total variance in drug expenditure across the five levels: treatment, patient, physician, market, and canton. Then, we included explanatory variables at each level and determined the contribution of these variables to explain the variance measured at each level. To assess the impact of each statistically significant variable on drug expenditure on an intuitive scale, we computed contrasts.

We found that more than two third of the drug expenditure variance was determined at the market level, and the variable having the greatest impact on drug expenditure at this level was market size (average expenditure decreased by 108 CHF in market with 10,000 vs 100,000 insured). Notice, unfortunately, that market size is not controllable per se as it depends mainly on the number of people with a specific illness and may not be used as tool to control drug expenditure. Notwithstanding this, Swiss drug authorities might still strive to negotiate lower prices with their providers together with other countries. Regarding market recentness, we found a sharp contrast between markets with new drugs and markets with older drugs (there was a difference of 104.8 CHF when the average age of drugs differed by 15 years). However, this contrast describes markets selling different drugs which may not be substitutable. Nevertheless, some markets may be substitutable and fostering the selling of old drugs proven to be efficacious may be one way to reduce expenditure. Of the four variables (HHI, number of mee-too, number of drugs, and number of brands) used in turn in different models to measure competition on the drug market none turned out to be significantly associated with expenditure. This is somewhat unexpected given economic theory claiming that in a free-market competition will lower prices.

The treatment level also had a significant influence on drug expenditure (about 20% of the expenditure variability was explained at this level). The number of prescribers had the largest impact (drugs prescribed by two physicians were 217 CHF more expensive than those prescribed by one single physician). This should not be over interpreted, however, as in practice most insured had (on average) between 1.0 to 1.1 prescribers and the effect of this variable would thus be much weaker. This effect might be explained by different mechanisms. First, we might assume that having multiple prescribers is a proxy for drugs for chronic conditions, which naturally require lengthy and more costly treatments. In this case, unfortunately, prohibiting consulting several physicians would not produce any economies. Alternatively, it also possible that patients solicit other physicians to lengthen the prescription duration that was refused by the first physician (e.g. sleeping pills); gatekeeping mechanisms might be effective to reduce costs in such cases. We found that the use of generics (-58.6 CHF) and older drugs (-36.2 CHF) were associated with lower expenditure. Generics are known to be cheaper than originals and incentives to use generics might contribute to cost reduction.

We found that older, substitutable, drugs were cheaper than newly released treatments, raising the question of the added value of drugs’ recentness. Between 2012 and 2014, 68.9% of 270 newly registered medications corresponded indeed to already known active substances [29]. This shows that most of the newly released medications consisted essentially in a modification of the presentation of the drugs (administration mode, dosage, number of pills, co-marketing of same substance) or in “me-too” drugs without great differences in efficacy. One option to curb this tendency could be to adopt a same fixed price per defined daily dose (DDD) for substitutable drugs among a same ATC 4^th^ level (generics and me-too drugs). This would provide incentives to convince physicians and patients to choose the most effective and comfortable drugs (substance, administration way, packaging, etc.) without pernicious effects on expenditure. This strategy makes sense given that physicians are often unaware of the actual cost of medications [30]. Another solution would consist in fixing prices per defined daily dose (DDD) for all products within a same market, which would not change the prices at short term but would at least stabilize them. Of course, innovative drugs (i.e. new substances) might increase expenses ([31] for instance), but this was not studied here, because our results were adjusted by markets (ATC4^th^ level). Finally, although Rx prescription should not depend on expenses’ aspects, the fact that drugs requiring a physician’s prescription tended to be associated with higher expenditure (+ 92 CHF) might suggest that marketing efforts were effective [32].

The three remaining levels were responsible for only 5% of the drug expenditure variability, showing that measures based on patients, physicians or cantons should not have a great impact on costs reduction. At the patient level, we found that elderly and patients with multi-morbid conditions might receive more costly drugs to avoid interaction for instance, confirming the results of previous studies [33]. The contrasts observed for the other patient-level variables (deductible and gender) were small. These results confirm the weak role of patients in choosing treatments, even if a greater involvement of them might be desirable [34]. Although the physician level was associated to less than 1.7% of the drug expenditure variance, variables that were introduced in our model at this level (i.e. type of physician and their propensity of using recently introduced drugs) explained half of it. Finally, it is interesting to see that regional disparities were relatively slight, contrarily to other geographic comparison of drug consumption in Switzerland [35].

The main finding of this study is that drug recentness was associated with an increase in drug expenditure, confirming former studies stating that pharmaceuticals recently introduced to the Swiss market generated higher costs [36]. In most economic sectors, new products enables the competitors to keep market shares, in a context of stable or decreasing prices. But, in the drug sector, it is a way to increase expenditure. Moreover, we found that several other aspects of the economic theory of perfect competition did not apply (e.g. more concurrence and higher deductibles were not associated with a decrease in drug expenditure). Such results are congruent with former researches on the absence of effect of the co-payment on rational use of medicines [37], and on the weak impact of financial incentives on prescribing medicines [38, 39]. They also provide evidence that the drug market cannot be considered as a “perfect market”, knowing that the patient is the customer, the decision is medical, the payment is not made by the insurance, the pharmaceutical industry sometimes has a dominant position, the entry of a new supplier into the market is not easy, and the substitution among products is not always possible [13].

Our study had some limitations. First, we assumed that physicians might substitute drugs in each market without damaging the efficacy of treatment. Even if this assumption should hold in most of the cases, physicians might have good reasons to choose a more costly drug, e.g. to provide a better comfort to the patient (administration mode for instance), or to avoid some side effects or drugs’ interactions [40], especially for multi-morbid patients [41]. However, we adjusted our results for age and co-morbidity index and only substitution among the same or similar substances were taken into account.

Second, our dataset was relatively old (i.e. 2006). There is no guarantee that the results obtained that year would be identical today. For this reason, this analysis should be seen as historical (or even pioneer), illustrating which kind of conclusions might be obtained with similar data and model. For instance, asserting that the variables linked to market and treatments (generic, etc.) explain respectively 76% and 20% of the variance of annual treatment costs provides an information that is crucial to know to take sensible decisions. Whereas it is well known that superficial innovations (co-marketing, packaging, etc.) might help imposing higher prices, it is also important to confirm it empirically and, eventually, to measure the magnitude of this effect. If our results were to be confirmed with updated data, it would indicate that it is probably more effective to act on the structure of the market than to target patients or doctors. An analysis with more updated data would also enable to take into account new aspects such as the emergence of healthcare networks or biosimilar drugs [42, 43].

Some drivers of drugs’ expenditures in 2006 Switzerland’s market might well still play an important role in today’s drug market, both markets displaying several similarities (e.g. many substitutable drugs, new products regularly introduced in the reimbursement list of drugs). However, we must also mention some changes that have occurred in 16 years, such as the apparition of new treatments against cancer and autoimmune diseases, an increased pressure of Federal authorities on prices since 2012, and a growing market share for generics. We could thus only encourage further research on this subject with updated data to see whether and to which extent results obtained here still apply, in Switzerland or in other countries, and we hope that this study will motivate stakeholders to make the required datasets available in the near future.

## Data Availability

The data that support the findings of this study were provided by insurers through the Swiss Federal Office of Public Health. The data used are not publicly available and was provided on a voluntary basis by the insurers, who required restrictions for certain specific research to prevent the data from being used against their interests (risk adjustment or other issues). For reproducibility purpose, data used in the statistical analysis are available from the authors upon reasonable request and with permission of the Swiss Federal Office of Public Health and insurers.
